# Biopsy-Proven Cerebral Parenchymal Involvement in IgG4-Related Disease: A Case Report and a Literature Review

**DOI:** 10.7759/cureus.98564

**Published:** 2025-12-06

**Authors:** Rui Nakajima, Nanami Takeda, Mai Okubo, Kurumi Asako, Hirotoshi Kikuchi, Koji Saito, Satoe Numakura, Yuko Sasajima, Hajime Kono

**Affiliations:** 1 Department of Internal Medicine, Teikyo University School of Medicine, Tokyo, JPN; 2 Department of Pathology, Teikyo University School of Medicine, Tokyo, JPN; 3 Department of Pathology, Jichi Medical University School of Medicine, Shimotsuke, JPN

**Keywords:** brain biopsy, brain parenchymal lesion, central nervous system involvement, glucocorticoid, igg4-related disease

## Abstract

We present a case of biopsy-confirmed IgG4-related disease (IgG4-RD) presenting as a solitary brain lesion despite normal serum IgG4 levels, along with a literature review of 12 cases. A woman in her 70s presented with seizures and impaired consciousness. Contrast-enhanced brain magnetic resonance imaging (MRI) revealed an enhanced lesion in the frontal lobe white matter. Endoscopic brain biopsy demonstrated perivascular infiltration of plasma cells within the brain parenchyma, with 25 IgG4-positive plasma cells per high-power field and an IgG4/IgG-positive cell ratio of >40%. The patient's serum IgG4 level remained within the normal range. A diagnosis of IgG4-RD was established, and prednisolone therapy was initiated. Follow-up MRI showed marked improvement in the lesion. A literature review identified 12 IgG4-RD cases with brain parenchymal lesions. The literature review revealed that normal serum IgG4 levels are not uncommon in this manifestation and that limb motor dysfunction is the most frequent symptom. In most cases, glucocorticoids are the mainstay of treatment. In conclusion, IgG4-RD can present as an isolated brain parenchymal lesion without elevated serum IgG4. Brain biopsy is critical for accurate diagnosis to differentiate it from other conditions, such as malignant lymphoma, and to guide appropriate therapy.

## Introduction

IgG4-related disease (IgG4-RD) is a systemic autoimmune disorder characterized by elevated serum IgG4 levels and infiltration of IgG4-positive plasma cells into various organs. The prevalence has been reported as 0.28 to 1.08 cases per 100,000 population in Japan, with a higher incidence in males [1]. Glucocorticoids are effective in many cases. This disease is now recognized to potentially involve nearly every organ system. Clinical manifestations are diverse, including pancreatitis, sclerosing cholangitis, dacryoadenitis, sialadenitis, ocular lesions, interstitial nephritis, retroperitoneal fibrosis, and lymphadenopathy [2,3]. Histopathologically, it is characterized by IgG4-positive plasma cell infiltration, storiform fibrosis, and obstructive venulitis, as demonstrated by biopsy of the affected organs [3]. Central nervous system (CNS) involvement in IgG4-RD has been reported in conditions such as hypophysitis and hypertrophic pachymeningitis; however, cases presenting with parenchymal brain lesions are exceedingly rare [4].

In this report, we present a case of IgG4-RD presenting with a brain parenchymal lesion confirmed by biopsy. We also review relevant literature and discuss our findings in the context of previous reports. We aim to contribute to future clinical practice by presenting the clinical features and therapeutic approach in this case report.

## Case presentation

**Figure 1:**
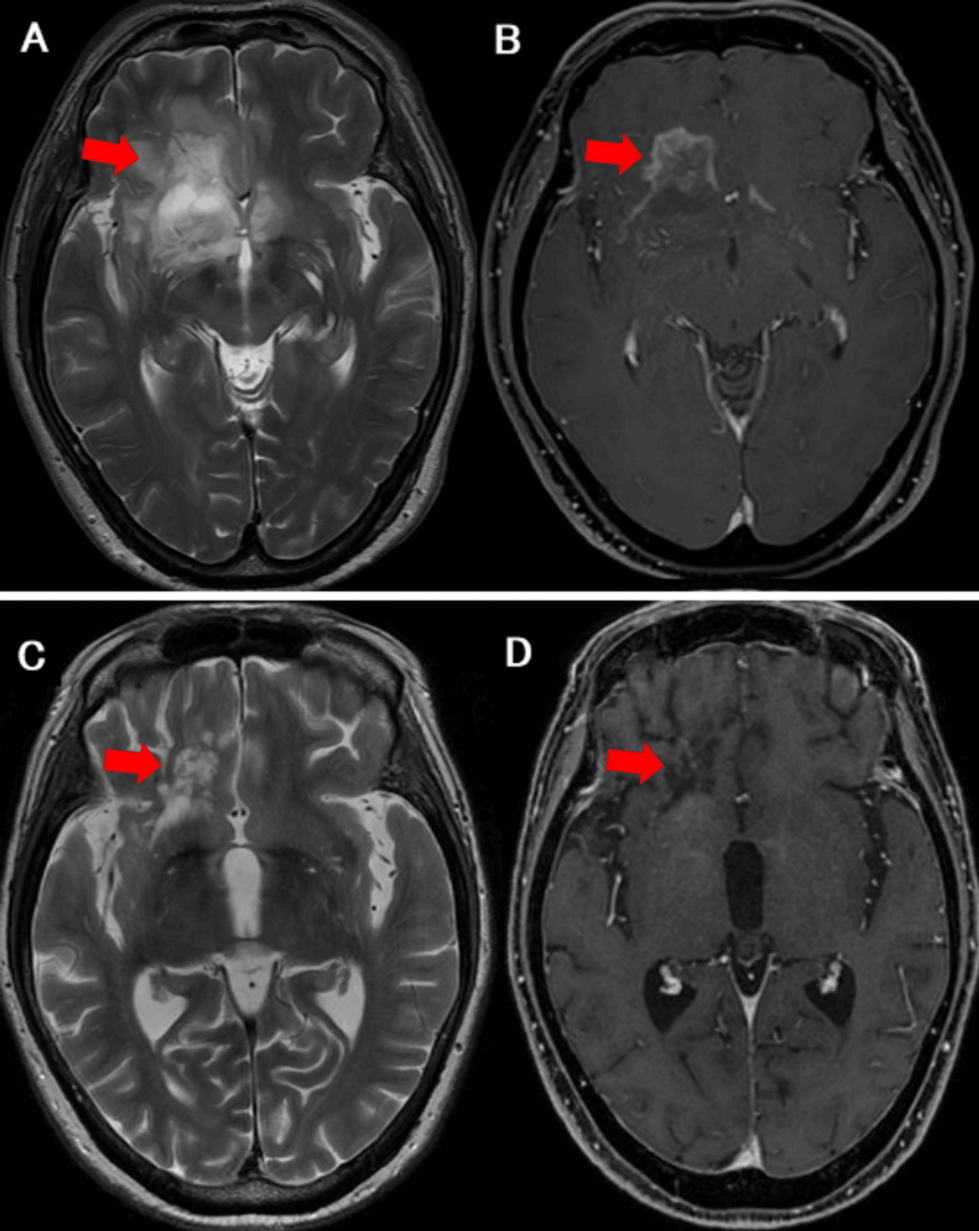
Contrast-enhanced brain magnetic resonance imaging before and after treatment A contrast-enhanced brain magnetic resonance imaging (MRI) showed a high-signal area (A) on the T2-weighted image and a corresponding enhancing lesion in the white matter of the frontal lobe (B). After 36 days of prednisolone therapy, a follow-up MRI showed marked improvement in the brain parenchymal lesion (C and D).

A woman in her 70s was admitted to our hospital because of generalized seizures and impaired consciousness. Her body temperature was 35.6℃, blood pressure was 177/121 mmHg, and pulse rate was 142 beats per minute. Except for inhalation therapy and oral theophylline administered for bronchial asthma over the past two decades, the patient had no significant medical history. Treatment with levetiracetam led to a rapid improvement in both seizures and consciousness. At the time, she showed right conjugate gaze deviation. After regaining consciousness, it completely resolved, and neurological findings were unremarkable. Furthermore, she was unaware of any distinct prodromal symptoms, including headache, before losing consciousness. Head computed tomography (CT) revealed a hypodense lesion in the right frontal lobe. Contrast-enhanced brain magnetic resonance imaging (MRI) revealed a corresponding enhancing lesion in the white matter of the frontal lobe (Figure 1B).

We present the main findings from the blood and urine examinations (Table 1). The incidentally detected hyponatremia was corrected at an appropriate rate. The serum IgG4 level was 42.4 mg/dL, which remained within the normal range. Immunoserological tests showed an elevated rheumatoid factor of 67.3 IU/mL (reference range: 0-15 IU/mL), while the antinuclear antibody level was <40-fold, and MPO-ANCA, PR3-ANCA, and anti-SS-A antibodies were negative. Serum angiotensin-converting enzyme levels were normal, whereas soluble interleukin-2 receptor levels were mildly elevated (931 U/mL).

**Table 1:** Laboratory findings on admission

Laboratory Findings	
Peripheral blood	
Red blood cells	431 × 10^4^ /μL
Hemoglobin	13 g/dL
Hematocrit	37.80%
Mean corpuscular volume	87.7 fL
White blood cells	17,500 /μL
Neutrophil	55%
Lymphocyte	29%
Monocyte	9%
Eosinophil	5%
Basophil	1%
Myelocyte	1%
Platelet	22.6 × 10^4^ /μL
Blood chemistry	
Total protein	7.6 g/dL
Albumin	4.5 g/dL
Total bilirubin	0.13 mg/dL
Aspartate transaminase	30 U/L
Alanine aminotransferase	12 U/L
Lactate dehydrogenase	331 U/L
Alkaline phosphatase	61 U/L
γ-glutamyl transpeptidase	18 U/L
Blood urea nitrogen	8.2 mg/dL
Creatinine	0.6 mg/dL
Uric acid	4.5 mg/dL
Na	115 mEq/L
K	3.9 mEq/L
Cl	79 mEq/L
C-reactive protein	0.32 mg/dL
Immunoserological tests	
Rheumatoid factor	67.3 IU/mL
IgG	1,648 mg/dL
IgA	173 mg/dL
IgM	132 mg/dL
IgE	5,332 IU/mL
IgG4	42.4 mg/dL
C3	128 mg/dL
C4	17 mg/dL
Antinuclear antibodies	<40×
Anti-SS-A antibody	1.3 U/mL
PR3-ANCA	<1.0 IU/mL
MPO-ANCA	<1.0 IU/mL
Angiotensin-converting enzyme (ACE)	7.8 U/L
Soluble interleukin-2 receptor (sIL-2R)	931 U/mL
Urinalysis	
pH	6.0
Specific Gravity	1.011
U-protein	(1+)
U-occult blood	(2+)
U-white blood cells	(-)
White blood cell cast	(-)
Red blood cell cast	(-)
Granular cast	(-)

**Figure 2:**
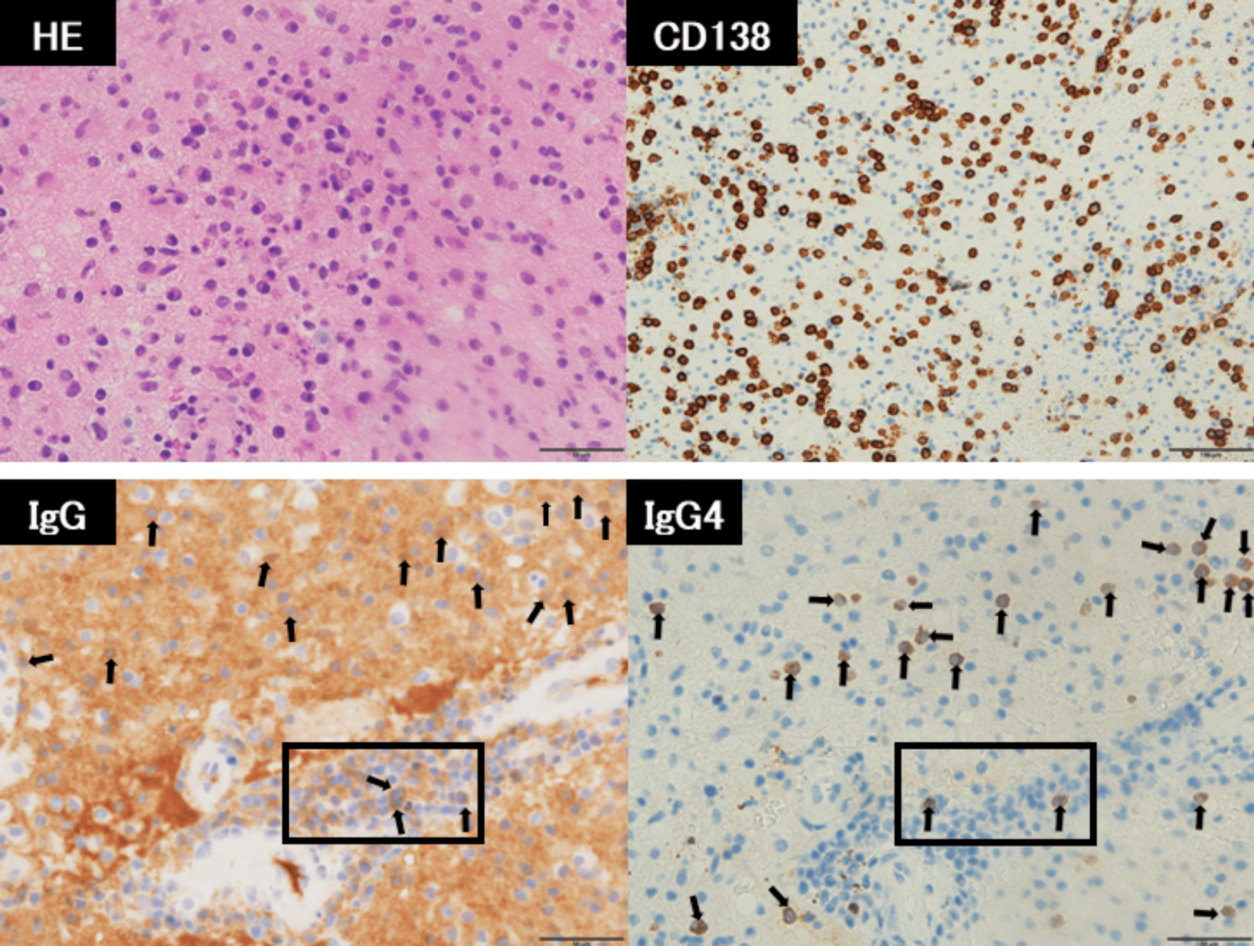
Histopathological images of the affected brain parenchymal lesion Histopathological examination revealed infiltration of plasma cells around blood vessels and within the brain parenchyma, with 25 IgG4-positive plasma cells per high-power field (HPF) and an IgG4/IgG-positive cell ratio >40%. Arrows were added for IgG and IgG4 staining to clarify the positive cells. IgG- and IgG4-positive cells were clearly observed within the framed area, which was selected for evaluating the IgG4/IgG-positive cell ratio.

Cerebrospinal fluid analysis revealed no pleocytosis, and bacterial and acid-fast bacilli cultures were negative. Regrettably, we could not measure IgG4 levels in the cerebrospinal fluid in this case. Fluorodeoxyglucose positron emission tomography/computed tomography (FDG-PET/CT) showed FDG accumulation in the supraclavicular lymph nodes, hilar and mediastinal lymph nodes, and right frontal lobe lesions. However, the supraclavicular lymph node lesion was small, and the hilar and mediastinal lymph nodes were not suitable for bronchoscopic biopsy; thus, a lymph node biopsy could not be performed. CT imaging revealed no other organ involvement suggestive of IgG4-RD, such as retroperitoneal fibrosis. An endoscopic brain biopsy was performed to diagnose brain parenchymal lesions and differentiate them from other conditions, like malignant lymphoma. Histopathological examination revealed infiltration of plasma cells around the blood vessels and within the brain parenchyma, with 25 IgG4-positive plasma cells per high-power field (HPF) and an IgG4/IgG-positive cell ratio >40% (Figure 2).

**Figure 3:**
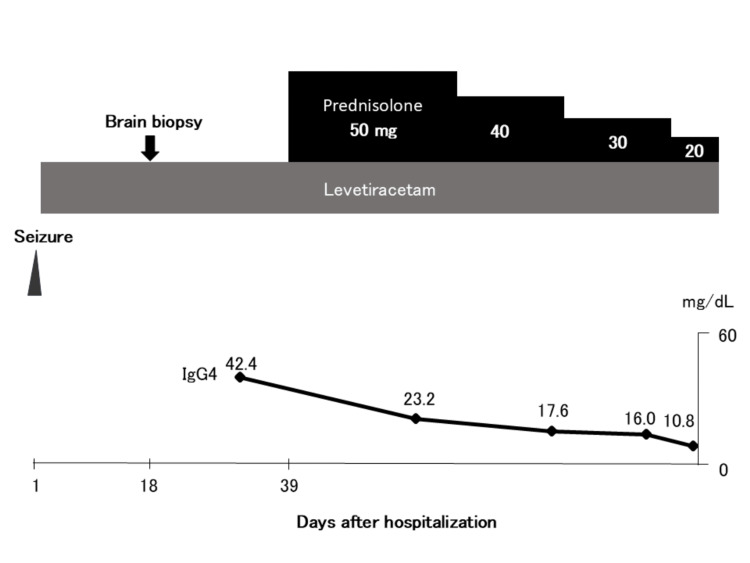
Clinical course

No histological evidence of malignancy or lymphoma was observed (no light-chain restriction; cyclin D1 and Bcl-2 were positive in a subset of cells; Ki-67 index was 1%-2%; Epstein-Barr virus-encoded small RNA in situ hybridization was negative). Based on these findings, a diagnosis of IgG4-RD with brain parenchymal involvement was made, and treatment with oral prednisolone (PSL) 50 mg daily was initiated. After 36 days of PSL therapy, follow-up MRI showed marked improvement in the brain parenchymal lesion (Figures 1C-1D). PSL was tapered to 20 mg by the time of discharge and further reduced to 7 mg at the most recent outpatient visit. Serum IgG4 levels remained within the normal range throughout the treatment course (Figure 3).

## Discussion

In this case, major differential diagnoses, including malignant lymphoma, sarcoidosis, primary brain tumors, and metastatic brain tumors, were ruled out based on blood tests, histopathological findings, and the clinical course. Although serum IgG4 levels were not elevated, the patient fulfilled the Japanese diagnostic criteria for “probable” IgG4-RD [5]. It is known that elevated serum IgG4 is not observed in all cases. Among cases clinically diagnosed with IgG4-RD based on biopsy, some reports indicate that serum IgG4 elevation was present in only 51% of cases [6]. A favorable therapeutic response to PSL further supported the diagnosis. Considering the overall clinical course, this case was deemed consistent with IgG4-RD with brain parenchymal involvement.

To identify similar cases, we searched the literature on PubMed using the keywords “IgG4-related disease” AND "brain parenchymal." Moreover, we conducted a review by examining the references cited in the retrieved papers as well. Brain parenchymal involvement in IgG4-RD is rare; however, several cases have been reported [7-18]. We conducted a literature review and identified 12 relevant cases (Table 2). Among the 13 patients with abnormal signals in the brain parenchyma on brain MRI, eight were male and five were female. Most patients presented between 50 and 70 years, consistent with the general trends observed in IgG4-RD [19], although the youngest patient was 14 and the oldest was 82 years old. Limb motor dysfunction (five cases) was the most common presenting symptom, followed by headaches (four cases). Other neurological manifestations included cognitive dysfunction, altered consciousness, seizures, diplopia, facial numbness, and ptosis.

**Table 2:** Characteristics of 13 cases presenting with brain parenchymal lesions GC: Glucocorticoid; IVMP: Intravenous methylprednisolone; RTX: Rituximab; MMF: Mycophenolate mofetil; P: Pachymeningitis; L: Leptomeningitis; W: White matter; G: Gray matter; M: Male; F: Female

Age/Sex	Affected area (W or G)	Involvement of meningitis (P or L)	Brain biopsy	Symptoms	Serum IgG4	Treatment (prednisolone equivalent amount)	IVMP	Prognosis	Involvement of other organs	Reference number
43/M	Left frontal lobe (W, G)	P	IgG4/IgG, 94%; IgG4-positive plasma cells, 72.4/HPF	Limb motor dysfunction (right arm weakness), Headache	Not elevated	GC (20 mg), Surgery	－	Improved	－	[7]
50s/M	Right frontal lobe (W), Bilateral temporal lobe (W)	－	IgG4/IgG: high (number not specified); IgG4-positive plasma cells >10/HPF	Cognitive impairment, Limb motor dysfunction (left hemiplegia)	Elevated (411 mg/dL)	GC (60 mg), RTX	＋	Improved	Autoimmune pancreatitis, sclerosing cholangitis, Mikulicz disease	[8]
58/F	Right frontal lobe (W, G)	P	IgG4/IgG, 60%; IgG4-positive plasma cells, 60/HPF	Limb motor dysfunction (left arm weakness)	Not elevated	GC (96 mg), Surgery	－	Improved	－	[9]
46/M	Temporal lobe (W, G)	P, L	Not done	Fatigue, Weight loss, Headache, Right-sided facial numbness, Diplopia on gaze to the right	Unspecified	GC (60 mg), Azathioprine	＋	Improved	－	[10]
58/F	Left cerebral hemisphere (W)	－	No specific findings	Consciousness disorder, Limb motor dysfunction (right-hand clumsiness), Speaking difficulty	Elevated (261 mg/dL)	GC (60 mg)	－	Improved	Sclerosing cholangitis	[11]
29/M	Left frontal lobe (W), Left parietal lobe (W)	－	IgG4/IgG 50%, IgG4-positive plasma cells 10-15/HPF	Headache, Memory loss	Elevated (3.04 g/L)	GC (100 mg), Cyclophosphamide	－	Improved	Autoimmune hepatitis	[12]
62/M	Left hypothalamus, Bilateral insular cortex (G), Putamen	－	Not done	Abdominal discomfort, Altered consciousness	Elevated (1.490 mg/dL)	GC (40 mg)	＋	Improved	Lymph nodes	[13]
61/M	Left frontal cortex (G), Subcortical white matter (W)	L	IgG4/IgG ≧40%, IgG4-positive plasma cells >10/HPF	Cognitive dysfunction, Emotional disinhibition	Not elevated	GC (25 mg)	＋	Improved	－	[14]
14/F	Cerebral parenchyma (W), Midbrain	－	Not done	Diplopia, Left eyelid ptosis, Right facial numbness, Limb motor dysfunction (right lower limb weakness)	Elevated (3.45 g/L)	GC (70 mg), MMF	＋	Improved	Enlarged lacrimal gland, lymph nodes	[15]
82/M	Right frontal lobe (W), Left temporal lobe (W)	－	IgG4/IgG 45%, IgG4-positive plasma cells >10/HPF	Seizure	Not elevated	GC (dose unspecified), Surgery	－	Improved	－	[16]
50s/F	Left temporal lobe (W, G)	P	IgG4/IgG 10%, IgG4-positive plasma cells >40/HPF	Headache, Focal seizures	Unspecified	RTX	－	Improved	－	[17]
44/M	Right frontal and parietal lobes (W, G)	P, L	IgG4-positive plasma cells >50 /HPF, IgG4/IgG >40%, Obliterative phlebitis	Left facial numbness, Seizure	Unspecified	Unspecified	Unspecified	Unspecified	－	[18]
70s/F	Right frontal lobe (W)	－	IgG4/IgG >40%, IgG4-positive plasma cells 25/HPF	Seizure, Impaired consciousness	Not elevated	GC (50 mg)	－	Improved	Lymph nodes	Our case

Serum IgG4 levels were elevated in five of the 10 cases for which data were available. Brain biopsy was performed in 10 of the 13 cases, and nine demonstrated significant pathological findings consistent with IgG4-RD (defined as IgG4-positive plasma cell infiltration >10/HPF and/or IgG4/IgG-positive cell ratio ≥40%). In the remaining three cases, biopsies of other organs provided supportive evidence of IgG4-RD.

Although IgG4-positive plasma cell infiltration is a characteristic finding, it is not specific to IgG4-RD and can occur in conditions such as multicentric Castleman's disease, rheumatoid arthritis, ANCA-associated vasculitis, and other immune-mediated conditions [3]. Storiform fibrosis and obstructive venulitis are considered more specific for IgG4-RD; however, they were identified in only one of 13 patients who underwent brain biopsy. As IgG4-RD is a chronic disease, its CNS manifestations may be detected relatively early when symptoms, such as impaired consciousness or motor dysfunction, prompt investigation and potentially precede the development of advanced fibrotic changes. Malignant lymphoma should be carefully excluded, as it may partially respond to glucocorticoid treatment, thereby misleading the diagnosis. Although brain biopsy remains essential for definitive diagnosis, its invasive nature and the technical expertise required may limit its feasibility in certain cases. In such situations, biopsies of other affected organs and careful imaging evaluation of the brain parenchymal lesions are crucial.

The optimal imaging follow-up interval for IgG4-RD with brain parenchymal involvement remains unclear. In our case, brain MRI was performed approximately every six months for monitoring purposes, and there has been no major recurrence over the past two years. Among the 13 cases reviewed, six involved organs other than the CNS, including autoimmune pancreatitis, sclerosing cholangitis, Mikulicz disease, autoimmune hepatitis, swollen lymph nodes, and lacrimal gland enlargement. Notably, six patients had concomitant pachymeningitis or leptomeningitis, suggesting that parenchymal lesions may have resulted from inflammation or vascular oedema spreading from these conditions. A similar hypothesis has also been proposed regarding CNS involvement in ANCA-associated vasculitis [20]. Lesion locations varied and included the frontal, temporal, and midbrain lobes. In cases associated with pachymeningitis or leptomeningitis, parenchymal abnormalities reside in areas adjacent to the affected meninges. In contrast, among the seven patients with isolated brain parenchymal lesions, six involved white matter. IgG4-RD primarily affects exocrine glands, such as the lacrimal and salivary glands, though lesions may also occur in various other organs. However, many aspects of its pathophysiology remain unclear, and the reasons for lesions developing in the CNS require further investigation.

Interestingly, prior studies have suggested that serum IgG4 levels are often normal in patients with IgG4-RD with predominant leptomeningeal involvement [14]. In our review, three of the six patients with pachymeningitis or leptomeningitis had normal serum IgG4 levels (with the remaining three cases having unspecified data). Notably, in cases of hypertrophic pachymeningitis associated with ANCA-associated vasculitis, ANCA was not detected in the serum but was detected in the cerebrospinal fluid [21]. Additionally, elevation of IgG4 levels in cerebrospinal fluid has been reported in hypertrophic pachymeningitis associated with IgG4-RD. This suggests that autoreactive B cells localized to the CNS are also activated in IgG4-RD [22].

CNS involvement in IgG4-RD is primarily treated with glucocorticoids, similar to management strategies for the involvement of other organs, and generally yields favorable outcomes. Among the 13 patients reviewed, 11 received glucocorticoid therapy, either intravenously or orally. Additional immunosuppressive agents included rituximab (two patients), mycophenolate mofetil (one patient), cyclophosphamide (one patient), and azathioprine (one patient). Surgical resection was performed for therapeutic or diagnostic purposes in three patients. Scientific evidence supporting the use of immunosuppressive agents with glucocorticoids is limited [23,24]. Rituximab, in particular, has been used in cases of glucocorticoid resistance, with a 77% success rate in a prospective, open-label trial, and may become a key therapeutic option for refractory cases in the future [25].

## Conclusions

In summary, we present a case of IgG4-RD with brain parenchymal involvement and review the existing literature. Cases of brain parenchymal involvement confirmed using brain biopsy remain exceedingly rare. The limited number of cases restricts fully understanding the pathophysiology or establishing evidence-based treatment strategies beyond glucocorticoids. This further highlights the need for the continued accumulation of clinical data to better understand the full spectrum of CNS manifestations of this disease.
